# Facilitating Anti-Cancer Combinatorial Drug Discovery by Targeting Epistatic Disease Genes

**DOI:** 10.3390/molecules23040736

**Published:** 2018-03-23

**Authors:** Yuan Quan, Meng-Yuan Liu, Ye-Mao Liu, Li-Da Zhu, Yu-Shan Wu, Zhi-Hui Luo, Xiu-Zhen Zhang, Shi-Zhong Xu, Qing-Yong Yang, Hong-Yu Zhang

**Affiliations:** 1 Hubei Key Laboratory of Agricultural Bioinformatics, College of Informatics, Huazhong Agricultural University, Wuhan 430070, China; qyuan@webmail.hzau.edu.cn (Y.Q.); liumengyuan2017@outlook.com (M.-Y.L.); lym17@webmail.hzau.edu.cn (Y.-M.L.); zhulinda@hotmail.com (L.-D.Z.); luozhihui@simm.ac.cn (Z.-H.L.); 2 School of Life Sciences, Shandong University of Technology; No. 12 Zhangzhou Road, Zibo 255049, China; wuyushanna@126.com (Y.-S.W.); sleevexz@sdut.edu.cn (X.-Z.Z.); 3 Department of Botany and Plant Sciences, University of California, Riverside, CA 92521, USA; shizhong.xu@ucr.edu

**Keywords:** combinatorial drug, drug target, GWAS, epistasis, 3D genome

## Abstract

Due to synergistic effects, combinatorial drugs are widely used for treating complex diseases. However, combining drugs and making them synergetic remains a challenge. Genetic disease genes are considered a promising source of drug targets with important implications for navigating the drug space. Most diseases are not caused by a single pathogenic factor, but by multiple disease genes, in particular, interacting disease genes. Thus, it is reasonable to consider that targeting epistatic disease genes may enhance the therapeutic effects of combinatorial drugs. In this study, synthetic lethality gene pairs of tumors, similar to epistatic disease genes, were first targeted by combinatorial drugs, resulting in the enrichment of the combinatorial drugs with cancer treatment, which verified our hypothesis. Then, conventional epistasis detection software was used to identify epistatic disease genes from the genome wide association studies (GWAS) dataset. Furthermore, combinatorial drugs were predicted by targeting these epistatic disease genes, and five combinations were proven to have synergistic anti-cancer effects on MCF-7 cells through cell cytotoxicity assay. Combined with the three-dimensional (3D) genome-based method, the epistatic disease genes were filtered and were more closely related to disease. By targeting the filtered gene pairs, the efficiency of combinatorial drug discovery has been further improved.

## 1. Introduction

Human complex diseases, especially cancer, are not caused by single pathogenic factors but by multiple factors, so the traditional “one drug, one target” therapeutic mode often yields limited effects on complex diseases [1]. Compared with single-component drugs, combinatorial drugs can not only target multiple disorder factors but also have many other advantages, such as synergies, reduced toxicity and delayed drug resistance [1,2,3,4]. According to the Drug Combination Database Version 2.0 (DCDB 2.0, http://www.cls.zju.edu.cn/dcdb/) [4], there have been 1363 combinatorial drugs collected, including 1033 investigational and 330 approved by the Food and Drug Administration (FDA). Therefore, combinatorial drugs have become a new trend for treating complex diseases. However, the traditional methods for combinatorial drug discovery are mainly experiment-based and may cause combinatorial explosion with increases in combination numbers, thus making them time-consuming and costly [5,6]. In addition, experiment-dependent studies are not able to explain the synergy mechanisms at the molecular level [1]. Thus, it is of great significance to develop theoretical methods that can direct combinatorial drugs in a reasonable way and make the components synergistic.

Medical genetics reveals the genotype–phenotype links in diseases and therefore provides critical information for drug discovery and drug repositioning [7,8,9,10]. Recently, genome wide association studies (GWAS) have identified a large number of disease-associated genes that are efficient sources of drug targets [9]. Recent studies show that targeting multiple disease-associated genes has greater therapeutic potential [11], and genes often exert functions through molecular interactions [12]. In addition, the therapeutic potential of chemical agents depends largely on the genetic links between targets and diseases [13]. The factors that can consolidate these links will enhance an agent’s medicinal potential. Thus, it is reasonable to speculate that targeting interacting disease-related genes (termed epistatic disease genes) may bring synergistic effects for disease control, and combinatorial drugs aimed at epistatic disease genes will have higher medicinal potential [14]. In this paper, we first combined drugs targeting synthetic lethality gene pairs for human tumors, which are similar to epistatic disease genes. The result showed that the combinatorial drugs that we screened are enriched with anticancer combinations, which validated our hypothesis. However, the source of synthetic lethality gene pairs is limited, so we then tried to identify epistatic disease genes from the GWAS dataset of breast cancer and demonstrated further that epistatic disease genes can be an important source of targets for combinatorial drugs.

The recent flourish in GWAS data has stimulated broad interest in the development of methodologies for searching gene–gene interactions on a genome-wide scale [14]. During these efforts, various algorithms, such as linkage disequilibrium (LD) and haplotype-based, data-filtering-based, data-mining-based, and machine-learning-based algorithms, have been applied [14]. Using one of these methods, resistance-associated gene pairs in *Mycobacterium tuberculosis* have been identified by calculating the interactions of single nucleotide polymorphism (SNP) pairs [15]. In this article, we tried to identify epistatic disease genes from the GWAS dataset of breast cancer using epistasis detection software. Through targeting the epistatic disease genes, combinatorial drugs were screened. Nine groups of these combinatorial drugs were incubated with MCF-7 cells, and five of them showed synergistical anti-breast cancer activity. Then, all the identified gene pairs were filtered with a three-dimensional (3D) genome dataset to strengthen the genetic links with diseases. Comparing combinatorial drugs targeting epistatic disease genes and the filtered genes, the latter were richer in anti-breast cancer combinations. In conclusion, we showed that taking epistatic disease genes as targets can narrow the search scope of disease-related targets and greatly improve the efficiency of combinatorial drug discovery.

## 2. Results and Discussion

**Figure 1:**
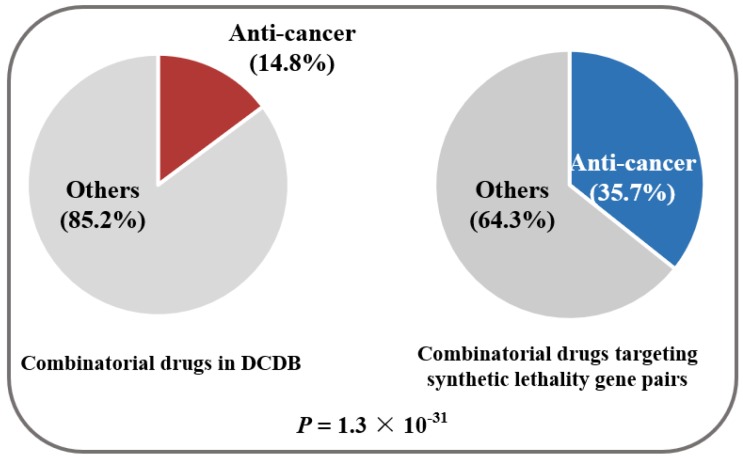
Medicinal potentiality analysis of the combinatorial drugs targeting synthetic lethality gene pairs. *p*-values were calculated using the hypergeometric test.

To perform this exploration, we first started with synthetic lethality genes. Synthetic lethality genes are gene pairs that lead to cellular or organismal death upon simultaneous mutations [15], which are considered to behave similarly to interacted genes. From the Synthetic Lethality Database (SynLethDB, https://labworm.com/tool/synlethdb), 19,952 synthetic lethality gene pairs for human tumors were collected with literature retrieval or theoretical prediction (available upon request) [15]. In a search of the Drug–Gene Interaction database (DGIdb, http://www.dgidb.org/), DrugBank (http://www.drugbank.ca) and the Therapeutic Target Database (TTD, https://db.idrblab.org/ttd/) [16,17,18], we identified combinatorial drugs that can target the synthetic lethality gene pairs. Through searching the DCDB [4], we found 342 documented combinations containing the predicted combinatorial drugs, 122 (35.7%) of which are efficient for treating cancer (Table S1). This ratio is significantly higher than the background ratio of anti-cancer combinatorial drugs in the DCDB [4] (14.8%, *p* = 1.3 × 10^−31^, hypergeometric test) (Figure 1). Therefore, it is concluded that these interacted genes are efficient sources of targets for combinatorial drugs.

Because of the limited source of synthetic lethality genes, we attempted to identify epistatic disease genes from a well-defined breast cancer GWAS dataset [19] using epistasis detection tools. Using the identified epistatic disease genes, we tried to demonstrate further the epistatic effects on the medicinal potential of combinatorial drugs. The breast cancer GWAS dataset that we used comprises 528,173 SNPs obtained from 1145 postmenopausal women of European ancestry with invasive breast cancer and 1142 control women [19] (available upon request). The assessment of population stratification was performed in a previous study [19]. To identify epistatic disease genes on a genome-wide scale, eight software packages were used in this study, including parallel multifactor dimensionality reduction (pMDR) [20], GBOOST [21], PLINK [22], FastEpistasis [23], SNPRuler [24], AntEpiSeeker [25], Ranger [26] and BEAM3 [27]. Starting from the GWAS dataset mentioned above [19], we evaluated the performance of eight genetic epistasis detection software packages in regard to their ability to identify interacting gene pairs. All eight software packages were run using a default configuration of each tool on the same workstation, and their performances are listed in Table 1. AntEpiSeeker [25] failed to complete the calculation in one month; Ranger [26] and BEAM3 [27] failed to identify any significantly associated SNP pairs; pMDR [20] and SNPRuler [24] could not identify significantly associated drug target pairs. In comparison, conventional methods based on regression algorithms, i.e., GBOOST [21], PLINK [22] and FastEpistasis [23], showed good performances.

**Figure 2:**
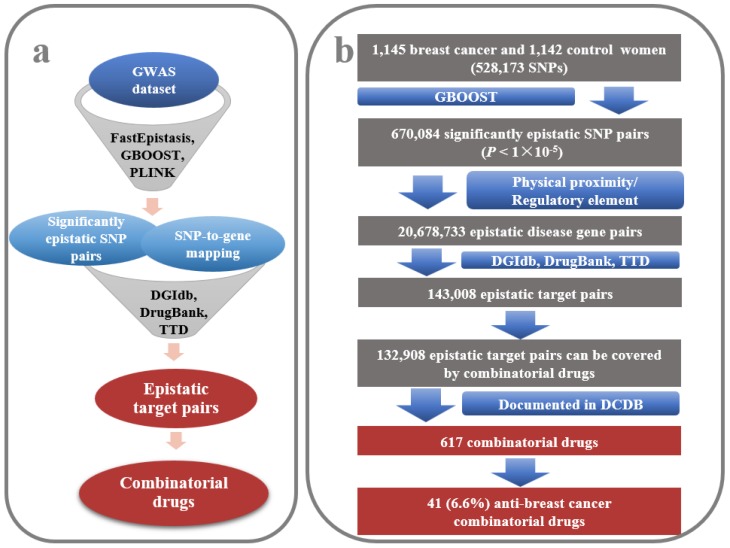
Pipeline for identifying epistatic target pairs in genome wide association studies (GWAS) and relevant combinatorial drugs. (**a**) The method for detecting significant epistatic target pairs and combinatorial drugs; (**b**) A case study by GBOOST. A total of 132,908 significantly epistatic target pairs can be hit by combinatorial drugs. The combinatorial drugs cover 617 of the combinations in the Drug Combination Database (DCDB, http://www.cls.zju.edu.cn/dcdb/), and 41 (6.6%) of them have clinical effects on breast cancer (*p* = 1.2 × 10^−6^, hypergeometric test).

**Figure 3:**
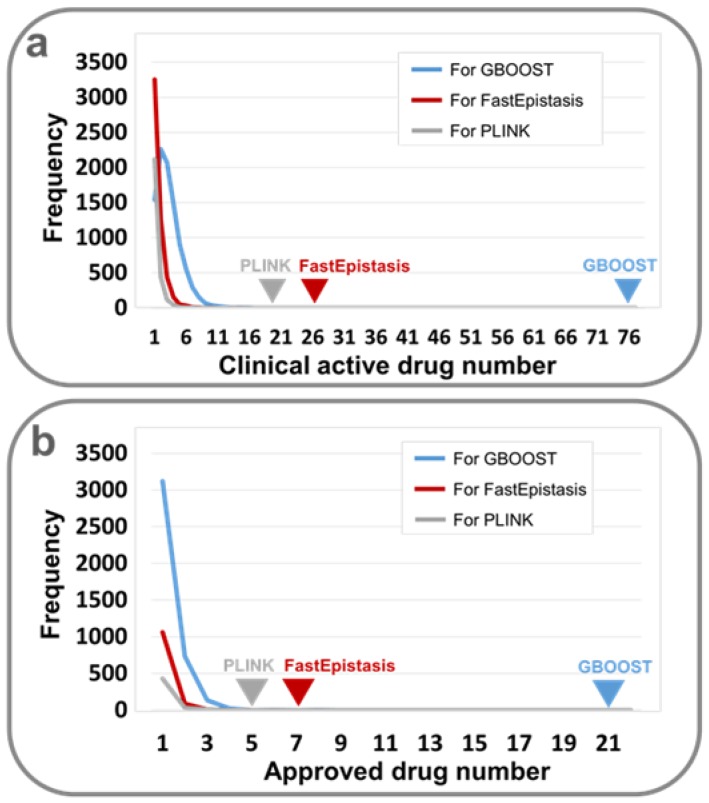
Frequency of clinically active/approved drugs hitting target pairs derived from GWAS: (**a**) for clinically active drugs; (**b**) for clinically approved drugs. Lines show the frequency of clinically active/approved drugs hitting target pairs derived from 10,000 random target combinations, where GBOOST, PLINK and FastEpistasis software were used to identify the epistatic target genes. Triangles indicate the number of active/approved drugs hitting epistatic target pairs calculated by GBOOST/PLINK/FastEpistasis software. It is evident that the epistatic target pairs are more promising as drug targets than random target combinations (*p* < 1 × 10^−4^, Permutation test).

GBOOST [21] yielded 670,084 significant epistatic SNP pairs (*p* < 1 × 10^−5^) (available upon request). Using the SNP-to-gene mapping method of Nelson et al. [28] and drug-target information derived from the databases DGIdb, TTD and DrugBank (Figure 2a) [16,17,18], 143,008 significant epistatic target pairs were identified (Figure 2b and Table S2). It is of interest to examine the functions of genes that commonly occurred in the target pairs. Disease-related genes were derived from the following nine databases: Genetic Association Database (GAD, http://geneticassociationdb.nih.gov) [29], Online Mendelian Inheritance in Man (OMIM, http://omim.org/) [30], Clinvar (http://www.ncbi.nlm.nih.gov/clinvar/) [31], Orphanet (http://www.orpha.net/consor/cgi-bin/index.php), GWASdb (http://jjwanglab.org/gwasdb) [32], NHGRI GWAS Catalog (http://www.ebi.ac.uk/gwas/) [33], DriverDBv2 (http://ngs.ym.edu.tw/driverdb/) [34], the records of Catalogue of Somatic Mutations in Cancer (COSMIC, http://cancer.sanger.ac.uk/cosmic) [35], and partial data from the Human Gene Mutation Database (HGMD, http://www.hgmd.cf.ac.uk/ac/index.php), which appeared in Wang et al.’s work [36] (available upon request). To obtain a sufficiently comprehensive list of breast cancer-related genes, the Unified Medical Language System (UMLS) [37] was used to standardize diseases and UMLS::similarity was used to measure disease similarity [38]. As a result, 2341 breast cancer-related genes were identified (Table S3). The 159 genes with the top 5% occurrence were collected, 46 (28.9%) of which were shown to be connected with breast cancer (Table S4), indicating a significant enrichment of breast cancer genes (3.9%, *p* = 3.57 × 10^−27^, hypergeometric test). Among the identified target pairs, it was shown that a small fraction (1.3%) have the ability to be targeted by 985 agents (Table S5). Seventy-six (7.7%) of the 985 agents exhibited clinical anti-breast cancer activity (*p* < 1 × 10^−4^, Permutation test) (Figure 3a) and 21 (2.1%) agents have been approved for breast cancer therapy (*p* < 1 × 10^−4^, Permutation test) (Figure 3b and Table 2) [17,18,39]. The results showed that the identified interacting target pairs were not random results, and they could be valuable targets for combinatorial drugs.

Furthermore, through searching the DCDB [4], it was found that 617 documented combinatorial drugs contain the drug pairs that target the epistatic target pairs, and 41 (6.6%) of them are efficient for treating breast cancer (Figure 3b and Table S6). This ratio is higher than the background ratio of anti-breast cancer combinatorial drugs in DCDB [4] (3.9%, *p* = 1.2 × 10^−6^, hypergeometric test) (Table 3). The analysis using PLINK [22] and FastEpistasis [23] yielded similar results (Table 3 and Tables S2–S6). Together, it seems that these three methods are effective for identifying the interacting gene pairs that are responsible for certain diseases on a genome-wide scale, and have the potential to be used to facilitate GWAS-based combinatorial drug discovery, in particular, compared with single locus-focused GWAS methods. In fact, from the breast cancer-associated loci originally identified from the GWAS data by Hunter et al. [19], we could not identify any anti-breast cancer drugs.

To further verify our hypothesis, nine groups of predicted combinatorial drugs by PLINK, GBOOST and FastEpistasis were screened randomly (Table S7). The six single drugs contained in the nine combinatorial drugs were first incubated with the human breast cancer cell line, MCF-7, with increasing concentrations for 48 h to evaluate their cytotoxic effects [40]. All the drugs showed dose-dependent cytotoxicity in MCF-7 cells, and the median effective doses of the drugs (Dm) analogous to the IC_50_ are shown in Figure S1 [40]. According to the Dm value ratios over 48 h, the drugs were then combined at a fixed dose ratio, and the dose–effect relationships for the nine combinatorial drugs are shown in Figure S2 (Table S7) [40]. 

Combination indices (CI) were calculated to evaluate the anti-breast cancer effects of the combinations. Five of nine groups—dasatinib+vorinostat (3:1), gefitinib+vorinostat (10:1), cladribine+dasatinib (5:1), gefitinib+dasatinib (5:1) and cladribine+gefitinib (1:1)—exhibited synergistic effects on anti-MCF-7 cells; in particular, the first three groups exerted strong synergistic anti-cancer effects (CI < 1) (Table S7) [40]. The other three groups—sorafenib + vorinostat (2:1), sorafenib + everolimus (1:2) and cladribine+sorafenib (6:1)—produced antagonistic effects, and gefitinib + sorafenib (5:1) exhibited slight antagonistic effects (CI > 1) (Table 4, Table S7) [40]. Additionally, the synergistic activities of dasatinib + vorinostat, cladribine+dasatinib and cladribine+gefitinib were analyzed in detail. The results of the analysis showed that these three combinatorial drugs exert synergistic anti-cancer effects on MCF-7 cells by inducing cell cycle arrest, reactive oxygen (ROS) production and apoptosis through mitochondrial-mediated endogenous pathways [41,42].

**Figure 4:**
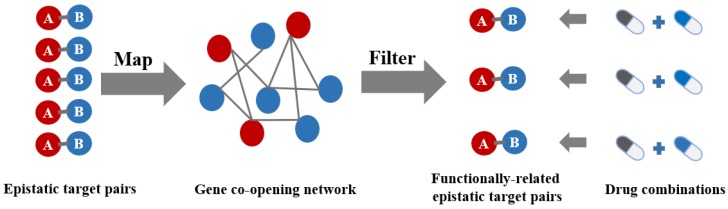
Pipeline for filtering epistatic target pairs by the co-opening network. Epistatic target pairs detected by three software packages were filtered with gene links that were considered to be functionally-related in the gene co-opening network. Then, a smaller number of functionally-related epistatic target pairs were left over. Finally, by targeting the filtered target pairs, drugs were combined after searching the Drug–Gene Interaction database (DGIdb, http://www.dgidb.org/), DrugBank (http://www.drugbank.ca) and the Therapeutic Target Database (TTD, https://db.idrblab.org/ttd/).

**Figure 5:**
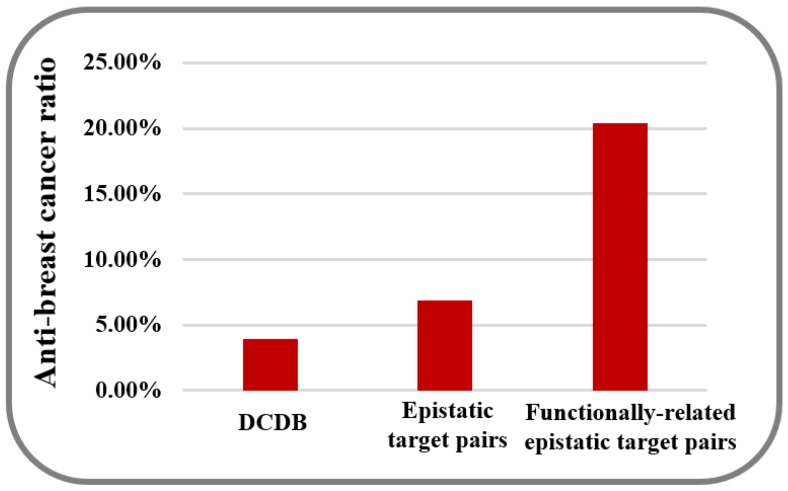
The clinically active ratio of combinatorial drugs targeting different target pairs. According to the DCDB, there are 53 (3.9%) combinatorial drugs that have been shown to have an anti-breast cancer effect in clinical trials. Targeting all the epistatic target pairs identified by GBOOST, PLINK and FastEpistasis, the predicted combinatorial drugs cover 651 combinations in the Drug Combination Database (DCDB, http://www.cls.zju.edu.cn/dcdb/), and 45 (6.9%) of the documented combinations were shown to be efficient for treating breast cancer. For targeting functionally-related epistatic target pairs, the predicted combinatorial drugs cover 49 combinations in DCDB and 10 (20.4%) of the documented combinations were shown to be efficient for treating breast cancer.

The results above fully indicate that targeting interacting disease genes can improve the efficiency of combinatorial drug discovery. At present, researchers have established a number of gene association maps that promote the identification of gene function [12]. Wenran et al. assumed that genes whose promoters are co-open for transcription factors on 3D structures tend to be co-active before transcription and exert functional interactions [43]. Therefore, they constructed a gene co-opening network that captured the correlation of the chromatin accessibility genes and validated the proposition that the genes were related to the same biological processes or diseases that tend to be linked in this network [43]. It is supposed that the genetic links between the functionally-related epistatic disease genes and breast cancer are stronger than the epistatic disease genes. To verify our hypothesis, we filtered all of the epistatic target pairs identified by GBOOST [21] PLINK [22] and FastEpistasis [23] with linked genes in co-opening network (Figure 4). As a result, we obtained 346 functionally-related epistatic target pairs whose numbers were reduced by three orders of magnitude (Table S8). Then, 49 combinatorial drugs in the DCDB were shown to contain the predicted combinations which target the target pairs after filtration, and 10 (20.4%) of them were shown to be clinically efficacious for anti-breast tumors (Table S9). This ratio is higher than the background ratio of anti-breast cancer combinatorial drugs in the DCDB [4] (3.9%, *p* = 8.6 × 10^−6^, hypergeometric test) and is also much higher than combinatorial drugs that simply target epistatic target pairs (6.9%, *p* = 9.8 × 10^−4^, hypergeometric test) (Figure 5). These results indicate that filtering epistatic target pairs with the co-opening network, constructed based on the 3D genome dataset, can screen out target pairs that are more closely related to disease than epistatic target pairs, so combinatorial drugs targeting these filtered target pairs are more likely to have medicinal potential.

## 3. Materials and Methods

### 3.1. Breast Cancer-Related GWAS Dataset

Sample genotypes from 2287 individuals of European ancestry provided by Hunter et al. [19] were used in this study. This dataset comprises 528,173 SNPs obtained from 1145 postmenopausal women with invasive breast cancer and 1142 control women [19]. Using the SNP-to-gene mapping method, described by Nelson et al. [28], 18,531 genes were identified, 1939 of which appeared in the list of 2341 breast cancer genes mentioned above (available upon request). This information was used to assess the significance of enriched breast cancer genes in the interacting target pairs.

### 3.2. Breast Cancer Genes

We collected 2341 breast cancer genes by combining nine sources: 933 breast cancer genes were identified in GAD [29], 99 breast cancer genes were identified in OMIM [30], 29 breast cancer genes were identified in Clinvar [31], 21 breast cancer genes were identified in Orphanet, 484 breast cancer genes were identified in the GWASdb & NHGRI GWAS Catalog [32,33], 972 breast cancer genes were identified in DriverDBv2 [34], 49 breast cancer genes were identified in COSMIC [35], and 77 breast cancer genes were identified in partial data from HGMD that appeared in Wang et al.’s work [36].

### 3.3. Information on Drugs

Drugs and their targets were collected from DGIdb, DrugBank, and TTD [16,17,18]. Of these, 10,941 drugs covering 3090 targets were collected from DGIdb [16], 4797 drugs covering 2244 targets were collected from DrugBank [17], and 5208 drugs covering 569 targets were collected from TTD [18]. By integrating the three datasets, we obtained 30,326 drug–target associations, including 14,125 drugs and 3231 target genes (available upon request). The indication information for the drugs were collected from DrugBank, TTD, and ClinicalTrials [17,18,39]. In total, we acquired 5716 drugs as well as their corresponding 90,549 drug–disease pairs (covering 665 types of diseases) and 15,965 drug–target pairs (covering 2289 target genes) (available upon request).

### 3.4. Information on Combinatorial Drugs

Combinatorial drugs and their indications were collected from the DCDB [4]. For each combination, the DCDB provides detailed annotations: indications, possible mechanisms, drug interactions between the components and the clinical stage. In this study, we used the current version, DCDB 2.0, which contains 1363 combinatorial drugs (330 approved and 1033 investigational).

### 3.5. Gene Co-Opening Network

The gene co-opening network was constructed by Wenran et al. [43]. They downloaded raw sequencing data from 628 DNase-Seq experiments from the ENCODE Project [12], Roadmap Epigenomics Project [44], and Pritchard lab [45]. Then, the transcription start sites (TSS) locations were mapped to the human genome. For each experiment, the chromatin accessible peaks were identified and used to build the co-expression network. The network they obtained consists of 11,461 genes and 271,900 interactions. The genes connected in the network are considered to be involved in the same biological process or disease.

### 3.6. Genetic Epistasis Detection in GWAS

In this study, the breast cancer-related GWAS dataset comprising 528,173 SNPs was used to detect genetic epistasis. All the SNPs with a missing genotype rate < 0.1, MAF > 0.05, a Hardy-Weinberg equilibrium (HWE) *p* > 0.001, and a pair-wise R^2^ < 0.8 were retained. In total, 498,847 SNPs were used for detecting genetic epistasis (available upon request). The eight most widely cited genetic epistasis detection software packages, including pMDR [20], GBOOST [21], PLINK [22], FastEpistasis [23], SNPRuler [24], AntEpiSeeker [25], Ranger [26], and BEAM3 [27], were used to detect SNP-SNP interactions. All eight software packages were run using the default configuration on a machine with an Intel Xeon E5-2630 CPU and an Nvidia Quadro K2000 GPU.

### 3.7. Permutation Test

To assess whether the identified interacting target pairs were random results, a permutation test was performed. Ten thousand samples were generated by random shuffling of significantly associated target pairs derived from GWAS. Then, we calculated the frequency of clinically active/approved drugs hitting target pairs derived from the 10,000 random target combinations. The frequency distributions were compared with the real frequencies of the clinically active/approved drugs, derived from the interacting target pairs calculated using the software GBOOST/PLINK/FastEpistasis [21,22,23].

### 3.8. Cytotoxicity Assays

#### 3.8.1. Cell culture and Reagents

MCF-7 cells were obtained from the China Center for Type Culture Collection (Wuhan, Hubei, China). Cells were cultured in RPMI-1640 medium (Gibco, Waltham, MA, USA) with 10% newborn bovine serum, in a humidified 5% CO_2_ incubator, at 37 °C. All drugs (purity > 98%) were purchased from Haoyuan Chemexpress Co., Ltd. (Shanghai, China) and dissolved in sterile dimethyl sulfoxide (DMSO) at 100 mmol/L (gefitinib, sorafenib and everolimus) or 200 mmol/L (cladribine, dasatinib and vorinostat ) stock solutions.

#### 3.8.2. Cytotoxicity Assays

Cytotoxicity assays of drugs on MCF-7 cells were determined by 3-(4,5-dimethylthiazol-2-yl)-2,5-diphenyltetrazolium bromide (MTT) assay (Sigma-Aldrich, St. Louis, MO, USA) [46]. Cells were seeded in 96-well plates at a density of 6 × 10^3^ cell/well. After overnight incubation, the cells were treated with drugs individually, and in combination, for 48 h, with 0.1% dimethyl sulfoxide DMSO as negative control. The drugs and combinatorial drugs were from stock solutions of each drug, and the maximal concentration of DMSO in media was less than 0.1% (*v*/*v*). About 10 µL of 5 mg/mL MTT solution was then added to the cells in 96-well plates and incubated for more than 4 h at 37 °C. In order to calculated inhibitory concentration 50% (IC_50_), the absorbance was detected at 492 nm using a Thermo Multiskan MK3 (Thermo Fisher, Waltham, MA, USA). According to the ratios of IC_50_, the ratios of combinatorial drugs were determined. All samples were measured in five replicates, and the experiments were repeated at least three times. The interaction between two drugs was analyzed according the median-effect principle, proposed by Chou and Talalay [47,48]. Dm and CI were calculated by CompuSyn software (version 1.0). A CI value represented synergism, additive effect, and antagonism, respectively, when it was below 1, equal to 1, and greater than 1, respectively.

## 4. Conclusions

Through working synergistically, combinatorial drug therapy can overcome disadvantages, such as poor efficacy, side effects, and drug resistance caused by a single drug [1,2,3,4]. However, selecting combinatorial drugs with desired activities is still a challenge [5,6]. Previous studies have revealed that genetic disease genes can provide valuable clues for drug activity prediction [7,8,9,10]. In addition, there are studies showing that targeting multiple disease genes may have more therapeutic potential [11], and the genes exert their functions by interaction [12]. Genetic links between the targets and diseases are critical factors for the treatment effects of combinatorial drugs [13]. Therefore, we speculated that targeting interacting genes that are disease-related will provide us with information on combinatorial drugs. In this study, by applying a therapeutic effects analysis to combinatorial drugs which target synthetic lethality genes or epistatic disease genes, we verified our hypothesis. The cell cytotoxicity assay indicated that there are five predicted combinatorial drugs showing synergistic anti-cancer effects on MCF-7 cells. Additionally, this study indicated that strengthened genetic links, produced by a 3D genome-based method, between target genes and diseases can also improve the medicinal potential of combinatorial drugs. This study revealed that epistatic disease genes are invaluable sources of drug targets. The method for detecting epistatic disease genes from a breast cancer GWAS dataset as targets can not only facilitate anti-breast cancer combinatorial drug discovery but can also be extended to combinatorial drug discovery for other complex diseases.

In addition, genetic epistasis is important in the pathogenesis of complex diseases and has recently attracted much attention [49,50,51]. However, due to various experimental, statistical and computational challenges, the true effectiveness of the methods for detecting epistasis is difficult to evaluate. In this work, using a well-defined breast cancer GWAS dataset, we assessed the performance of eight well-known genetic epistasis detection tools based on their drug enrichment efficiency. The results showed that the regression algorithm-based methods, such as GBOOST [21], PLINK [22] and FastEpistasis [23], performed consistently better than the other methods. Together, this study not only demonstrates the potential of epistatic disease genes as drug targets but also suggests a chemical biological method for evaluating epistatic disease gene-detecting methods.

## Figures and Tables

**Table 1:** Performance of software in identifying interacting single nucleotide polymorphism (SNP) pairs and gene pairs.

Software	Model	Version	Cost (Days) ^9^	SNP Pairs	Gene Pairs
GBOOST ^1^	Regression	-	<1	670,084	143,008
PLINK ^2^	Regression	1.9	<1	427,444	14,850
FastEpistasis ^3^	Regression	2.05	<1	498,482	48,189
pMDR ^4^	Data Mining	3.0.2	<1	500	0
AntEpiSeeker ^5^	Data Mining	1	>30	0	0
SNPRuler ^6^	Machine learning	-	~21	2	0
Ranger ^7^	Machine learning	0.5.0	~2	0	0
BEAM3 ^8^	Beyesian	1	~9	0	0

^1^ downloaded from http://bioinformatics.ust.hk/. ^2^ downloaded from http://www.cog-genomics.org/plink/1.9/. ^3^ downloaded from http://www.vital-it.ch/software/FastEpistasis. ^4^ downloaded from https://ritchielab.psu.edu/software/mdr-download. ^5^ downloaded from http://nce.ads.uga.edu/~romdhane/AntEpiSeeker/index.html. ^6^ downloaded from http://bioinformatics.ust.hk/. ^7^ downloaded from https://cran.r-project.org/web/packages/ranger/. ^8^ download from http://sites.stat.psu.edu/~yuzhang/. ^9^ by DELL T7600 Workstation with an Intel Xeon E5-2630 CPU and a Nvidia Quadro K2000 GPU.

**Table 2:** Clinically active ratio/approval ratio of drugs targeting epistatic target pairs calculated by PLINK/GBOOST/FastEpistasis.

Software	Clinically Active Ratio ^1^/*P* ^2,3^	Approval Ratio ^1^/*P* ^2,3^
GBOOST	76/985 (7.72%)/*P* < 1 × 10^−4^	21/985 (2.13%)/*P* < 1 × 10^−4^
PLINK	20/181 (11.05%)/*P* < 1 ×1 0^−4^	5/181 (2.76%)/*P* < 1 × 10^−4^
FastEpistasis	26/364 (7.14%)/*P* < 1 × 10^−4^	7/364 (1.92%)/*P* < 1 × 10^−4^

^1^ derived from target pairs calculated by PLINK/GBOOST/FastEpistasis. ^2^ derived from 10,000 random target combinations where software PLINK/GBOOST/FastEpistasis were used to identify the interacting target genes. ^3^ calculated by permutation test

**Table 3:** Clinically active ratio of combinatorial drugs targeting epistatic target pairs.

Software	Clinically Anti-Breast Cancer Ratio ^1^	Background Ratio ^1^	*p* ^2^
GBOOST	41/617 (6.6%)	53/1363 (3.9%)	1.2 × 10^−6^
PLINK	31/270 (11.5%)	53/1363 (3.9%)	8.0 × 10^−11^
FastEpistasis	36/355 (10.1%)	53/1363 (3.9%)	2.5 × 10^−10^

^1^ derived from Drug Combination Database (DCDB, http://www.cls.zju.edu.cn/dcdb/). ^2^ calculated by hypergeometric test

**Table 4:** Combination index of combinatorial drugs targeting epistatic genes.

Combinatorial Drugs	Combination Index ^1^	Software
Dasatinib + Vorinostat	0.439	BOOST/FastEpistasis
Gefitinib + Vorinostat	0.502	BOOST/FastEpistasis
Cladribine + Dasatinib	0.539	BOOST/FastEpistasis
Dasatinib + Gefitinib	0.628	BOOST/PLINK
Cladribine + Gefitinib	0.723	BOOST
Gefitinib + Sorafenib	1.288	BOOST/PLINK
Cladribine + Sorafenib	>1	BOOST/FastEpistasis
Everolimus + Sorafenib	>1	BOOST/PLINK
Sorafenib + Vorinostat	>1	BOOST/PLINK/FastEpistasis

^1^ Combination index > 1: synergistic effect; Combination index < 1: antagonistic effect

## Supplementary Materials

The following are available online, Figure S1: Anti-breast cancer effects of the six drugs contained in nine predicted combinatorial drugs; Figure S2 Synergistic anti-breast cancer effects of nine predicted combinatorial drugs. Table S1: Combinatorial drugs targeting synthetic lethality gene pairs, Table S2: Breast cancer-associated epistatic gene pairs calculated by software PLINK/GBOOST/FastEpistasis (*p* < 1 × 10^−5^), Table S3: Breast cancer-associated genes, Table S4: Genes with top 5% occurrence in epistatic gene pairs calculated by software PLINK/GBOOST/FastEpistasis (*p* < 1 × 10^−5^), Table S5: Drugs targeting epistatic target pairs calculated by software PLINK/GBOOST/FastEpistasis (*p*-value < 1 × 10^−5^), Table S6: Combinatorial drugs targeting epistatic gene pairs calculated by software PLINK/GBOOST/FastEpistasis (*p* < 1 × 10^−5^), Table S7: Combinatorial drugs screened for cell cytotoxicity assay, Table S8: Functionally-related epistatic gene pairs filtered by gene co-opening network, Table S9: Combinatorial drugs targeting functionally-related epistatic gene pairs.
